# Heterogeneity of quaternary structure of glucosamine-6-phosphate deaminase from *Giardia lamblia*

**DOI:** 10.1007/s00436-014-4174-4

**Published:** 2014-10-19

**Authors:** Karolina Kwiatkowska-Semrau, Justyna Czarnecka, Marek Wojciechowski, Sławomir Milewski

**Affiliations:** Department of Pharmaceutical Technology and Biochemistry, Gdańsk University of Technology, 11/12 Narutowicza Str., 80-233 Gdańsk, Poland

**Keywords:** *Giardia*, Glucosamine-6-phosphate deaminase, NagB, Quaternary structure

## Abstract

The oligoHis-tagged versions of glucosamine-6-phosphate deaminase from *Giardia lamblia* (*Glm*NagB-HisN, *Glm*NagB-HisC) were constructed and purified to hear homogeneity, and their kinetic and structural properties were compared to those of the wild-type enzyme (*Glm*NagB). Introduction of the oligoHis tag at the *Glm*NagB C-terminus resulted in almost complete loss of the catalytic activity, while the catalytic properties of *Glm*NagB-HisN and *Glm*NagB were very similar. The recombinant and wild-type enzyme exhibits heterogeneity of the quaternary structure and in solution exists in three interconvertible forms, namely, monomeric, homodimeric, and homotetrameric. Although the monomeric form is prevalent, the monomer/dimer/tetramer ratios depended on protein concentration and fell within the range from 72:27:1 to 39:23:38. The enzyme is fully active in each of the oligomeric structures, efficiently catalyzes synthesis of *D*-glucosamine-6-phosphate from *D*-fructose-6-phosphate and ammonia, and its activity is not modified by GlcNAc6P, UDP-GlcNAc, or UDP-GalNAc. GlcN6P deaminase of *G. lamblia* represents a novel structural and functional type of enzyme of the NagB subfamily.

## Introduction

NagB (glucosamine-6-phosphate deaminase; 2-amino-2-deoxy-*D*-glucose-6-phosphate aminohydrolase (ketol-isomerizing); EC 3.5.99.6, formerly EC 5.3.1.10) is in most organisms a catabolic enzyme and performs the isomerization and deamination reactions that transform *D*-glucosamine-6-phosphate (GlcN6P) to *D*-fructose-6-phosphate (Fru6P), with the concomitant release of ammonia. The reaction is basically reversible, but formation of GlcN6P by NagB is usually possible only at high substrate concentrations, so that under physiological conditions, the enzyme catalyzes a catabolic reaction (Calcagno et al. 1984). A notable exception to this rule is glucosamine-6-phosphate deaminase of *Giardia lamblia* (syn. *Giardia intestinalis*, *Giardia duodenalis*), a flagellated unicellular protozoan, which is the common infective parasite of a number of vertebrates. *G. lamblia* lacks GlcN6P synthase, an enzyme responsible for GlcN6P formation in an overwhelming majority of organisms (Milewski 2002), and this task is accomplished by its NagB (Adam 2001).

GlcN6P deaminase is an ubiquitous enzyme and is absent only from several eukaryotic lineages and plants (Álvarez-Añorve et al. 2011). Data accumulated so far indicate that there are three molecular versions of this enzyme, differing by quaternary structure and mechanisms of activity regulation. One of these versions, represented by *Escherichia coli* and mammalian NagB, is a homohexameric protein, activated by an allosteric effector, *N*-acetyl-d-glucosamine-6-phosphate/GlcNAc6P (Oliva et al. 1995; Arreola et al. 2003). On the other hand, GlcN6P deaminase of gram-positive bacteria including *Bacillus subtilis* and *Streptococcus mutans* is monomeric, and no physiological activator/inhibitor of this enzyme version has been identified (Vincent et al. 2005; Liu et al. 2008). Despite the structural and functional differences, both versions share a high homology of their primary sequences. A third type of protein demonstrating GlcN6P synthase activity was found in archea, has a homodimeric structure, demonstrates little homology to the mesophilic versions of NagB, but closely resembles the isomerase domain of GlcN6P synthase (Kim et al. 2007; Tanaka et al. 2005).

GlcN6P deaminase of *G. labmlia* (*Glm*NagB) seems to be different from their already described and characterized counterparts in other organisms, due to its specific, crucial role in the life cycle and metabolism of the parasite. The cyst of *G. lamblia* is relatively inert, allowing prolonged survival for a long period under harsh environmental conditions, particularly in cold water, mostly due to the rigid structure of its cell wall composed of carbohydrates and proteins. A unique carbohydrate component of the cell wall is a polymer of *N*-acetyl-d-galactosamine, synthesized in the inducible pathway, where GlcN6P deaminase catalyzes the first committed step. Although in *G. lamblia*, two genes encoding putative GlcN6P deaminase were identified, namely, *GPI1* and *GPI2*, only the former is functionally expressed (van Keulen et al. 1998). Expression of the *GPI1* gene (now called *GN6PI*) is ∼45 times higher during encystment than the basal level (Eligio-García et al. 2011). A similar increase is observed for the intracellular GlcN6P level (Sener et al. 2004), which reflects enhanced GlcN6P deaminase activity. All these changes appear when *Giardia* trophozoites encyst in the presence of bile (Eligio-García et al. 2011). Interestingly, a similar phenomenon of differential expression of homologous genes was also noted for some other genes of *G. lamblia*, for example, those encoding glyceraldehyde 3-phosphate dehydrogenase (Yang et al. 2002). The anabolic role of GlcN6P deaminase and lack of GlcN6P synthase was also observed in the case of some other human pathogenic protozoans, like *Entamoeba histolytica* and *Entamoeba invadens*, containing chitin in their cyst cell walls (Aguilar-Díaz et al. 2010).

Structural information on *G. lamblia* GlcN6P deaminase is very limited. Steimle et al. (1997) described some basic data for the native enzyme purified to near homogeneity in a multistep procedure. Those data suggested a monomeric structure of the enzyme and presence of its two isoforms, differing by the pI values.

It was postulated that enzymes of cell wall biosynthesis pathways could be targeted by potential antiprotozoal drugs (Spindler et al. 1990). The anabolic GlcN6P deaminase was indicated as one of the potential targets for antiparasitic chemotherapy (Aguilar-Díaz et al. 2011), which was confirmed by results of the gene silencing experiments (Samanta and Ghosh 2012). It seems interesting therefore to find out a molecular basis of an apparent uniqueness of this enzyme. In this paper, we present results of our studies on construction, overexpression in *E. coli*, purification, and characterization of oligoHis-tagged versions of *Glm*NagBp.

## Material*s* and methods

### Bacterial strains and growth conditions

The *E. coli* TOP 10F′ strain from Invitrogene was used in all cloning procedures. The *E. coli* BL21(DE3) pLysS strain from Novagen was used for the overexpression of the wild-type *Glm*NagB and its oligoHis-tagged versions. Bacterial strains were cultured at 37 °C on LA solid medium [1.0 % (*w*/*v*) NaCl, 1.0 % (*w*/*v*) tryptone, 0.5 % yeast extract, and 1.5 % (*w*/*v*) agar] and in LB liquid medium [1.0 % (*w*/*v*) NaCl, 1.0 % (*w*/*v*) tryptone, and 0.5 % yeast extract] supplemented with 0.1 mg/ml ampicillin.

### Plasmids, enzymes, and other materials

The pUC5-*Glm*NagB plasmid containing the *GPI1* (*GN6PI*) gene cloned from the *G. lamblia* MR4 strain (Van Keulen et al. 1998) was a generous gift of Dr. Harry van Keulen, Department of Biological, Geological and Environmental Sciences, Cleveland State University, Cleveland, OH. Other plasmids used were the following: pET15b (Novagen) and pETTopo from Champion pET Directional TOPO Expression Kits (Invitrogen). Restriction and modification enzyme DNA, molecular weight markers, and protein weight markers were purchased from Fermentas, Lithuania. DNA polymerase was from DNA-Gdańsk, Poland. T4 DNA ligase was from Epicentre.

Antibiotics, isopropyl-β-d-thiogalactopyranoside (IPTG), Fru6P, GlcN6P, and other reagents were purchased from Sigma. Purification of oligoHis-tagged proteins was performed on Ni^2+^-ion-dependent adhesion (IDA) agarose (His Bind Resin, Novagen). The wild-type enzyme was purified by ion exchange chromatography on the ResourceQ column.

### DNA manipulations

Isolation of plasmid DNA was carried out according to the protocol of the Plasmid Mini kit (A&A Biotechnology). DNA fragments were isolated from agarose gels following the standard procedure of the DNA Gel-Out kit (A&A Biotechnology). DNA purification after enzyme treatment was performed according to the instructions in the DNA Clean-up kit (A&A Biotechnology). DNA digestion with restriction enzymes was carried out according to the enzyme supplier’s instructions. DNA fragments were ligated, and *E. coli* cells were prepared and transformed according to the standard methods (Sambrook et al. 1989).

### Construction of bacterial expression plasmids

The recombinant expression plasmid encoding the wild-type GlcN6P deaminase of *G. lamblia* (pETTopo-*Glm*NagB) was constructed by PCR amplification from the pUC5-*Glm*NagB plasmid as a template, with the following primers: 5′-CACCATGCCGTCCATCCACGTC-3′ (underlined sequence needed for further procedures) and 5′-GGATCCATTCACGTGTTTAAGCTTTTGC-3′. The purified PCR product was then introduced into pETTopo using The Champion pET Directional TOPO Expression Kit (Invitrogen).

The recombinant gene encoding *G. lamblia* GlcN6P deaminase with C-terminal His_6_ fusion (pETTopo-*Glm*NagB-HisC) was constructed by PCR amplification from the pUC5-*Glm*NagB plasmid as a template, with the following primers: 5′-CACCATGCCGTCCATCCACGTCTCC-3′ (underlined sequence needed for further procedures) and 5′-CCTTA**ATGATGATGATGATGATG**AGCTTTTGCAGC-3′ (sequence introducing hexaHis is bold).

The recombinant gene encoding *G. lamblia* GlcN6P deaminase with N-terminal His_6_ fusion (pET15b-*Glm*NagB-HisN) was constructed by PCR amplification from the pUC5-*Glm*NagB plasmid as a template. Addition of a 5′-*Nde*I restriction site was done using the forward primer (underlined sequence encodes restrictions sites of the enzyme) 5′-ATCACATATGCCGTCCATCCACGTCTCC-3′, and a 3′-*BamH*I restriction site was generated using a reverse primer 5′-ATAGAGGATCCATTCACGTGTTTAAGCTTTTGCAG-3′. The amplified DNA was digested with *Nde*I and *BamH*I and inserted into the expression vector by ligation to the corresponding site of the *Nde*I/*BamH*I-digested pET15b vector.

The identity of recombinant plasmids was confirmed by restriction analysis and DNA sequencing.

### Protein expression

*E. coli* BL21(DE3) pLysS cells, transformed with pETTopo-*Glm*NagB, pETTopo-*Glm*NagB-HisC, or pET15b-*Glm*NagB-HisN expression plasmid, were grown overnight in the LB liquid medium supplemented with ampicillin, at 37 °C. Sample of this culture (10 ml) was then transferred to 1 l of a fresh LB broth containing ampicillin, and cell suspension was grown at 37 °C. Expression was induced by the addition of 1 mM IPTG to the cultures grown to OD_600_≈0.5 and incubation was continued for another 5 h. Cells were harvested by centrifugation at 3,000×*g* for 20 min at 4 °C.

### Purification of the wild-type *Glm*NagB

The wild-type *Glm*NagB was purified by ion exchange chromatography. The bacterial pellet was suspended in buffer A (25 mM KH_2_PO_4_/K_2_HPO_4_, pH 7.0, 0.5 mM PMSF, 1 mM DTT, 1 mM EDTA), and the cells were disrupted by sonication (3 × 30-s bursts with 30-s intervals at a power setting of 30 W, using a Branson sonifier 250) on ice. The lysate was centrifuged at 10,000×*g* for 20 min at 4 °C. DNA was removed from the supernatant by addition of streptomycin sulfate solution to the final concentration of 1.1 %. The sample was stirred on ice during the entire procedure and 15–30 min afterward. The resulting mixture was centrifuged for 20 min (10,000×*g* at 4 °C), and the supernatant was collected. The extract after streptomycin sulfate precipitation was treated with a saturated ammonium sulfate solution to the final concentration of 60 %. The suspension was stirred on ice during the entire procedure and 15–30 min afterward and then centrifuged for 20 min (10,000×*g* at 4 °C). The supernatant was discarded, and the precipitate was dissolved in ∼10 ml of buffer A supplemented with 40 mM NaCl and 10 mM MgCl_2_. After complete dissolution, a 50 % solution of polyethylene glycol 4,000 was added to a final concentration of 10 %. The suspension was stirred on ice during the entire procedure and 15–30 min afterward and then centrifuged for 20 min (10,000×*g* at 4 °C). The supernatant was discarded, and the precipitate was dissolved in 5–10 ml of buffer A supplemented with 40 mM NaCl and 10 mM MgCl_2_. The solution was loaded on ResourceQ FPLC 6-ml column equilibrated with buffer B (20 mM KH_2_PO_4_/K_2_HPO_4_, pH 7.0). The column was washed with 20–30 ml of buffer B, and elution was performed with a linear 0–1 M NaCl gradient in buffer C (20 mM KH_2_PO_4_/K_2_HPO_4_, 1 M NaCl pH 7.0). The elution rate was 5 ml/min. Active fractions were pooled and concentrated by ultrafiltration using Amicon® Ultra-15″ Centrifugal Filter Units (10-kDa cut-off limit) at 2,500×*g* at 4 °C, until the protein concentration reached approximately 2 mg/ml.

### Purification of oligoHis-tagged proteins

*Glm*NagB-HisC and *Glm*NagB-HisN were purified by metal affinity chromatography. The purification of these recombinant proteins was performed similarly. The bacterial pellet was suspended in the buffer D (20 mM Tris/HCl, pH 8, 500 mM NaCl, 5 mM imidazole, 0.1 % Tween 20, and 1 mM phenylmethylsulfonyl fluoride/PMSF/), and the cells were disrupted by sonication (3 × 30-s bursts with 30-s intervals at a power setting of 30 W, using a Branson sonifier 250) on ice. The lysate was centrifuged for 20 min (10,000×*g* at 4 °C). The supernatant (crude extract) was applied to a Ni^2+^-IDA agarose column, pre-equilibrated with the buffer D. The column was then washed with the same buffer, followed by washing with the buffer E (20 mM Tris/HCl, pH 8, 500 mM NaCl, 50 mM imidazole, 0.1 % Tween 20, and 1 mM PMSF). The oligoHis-tagged protein was eluted with buffer F containing 200 mM imidazole (20 mM Tris/HCl, pH 8, 500 mM NaCl, 200 mM imidazole, 0.1 % Tween 20, and 1 mM PMSF).

For further assays, the fraction collected was concentrated by ultrafiltration using Amicon® Ultra-15″ Centrifugal Filter Units (10-kDa cut-off limit; Millipore, 2,500×*g* at 4 °C) several times, until the protein concentration reached approximately 2 mg/ml. The buffer was then exchanged for buffer A (25 mM KH_2_PO_4_/K_2_HPO_4_, pH 7.0, 0.5 mM PMSF, 1 mM DTT, 1 mM EDTA) using the HiTrap™ Desalting Columns.

### Enzyme activity determination

GlcN6P deaminase activity in both aminating and deaminating direction was assayed colorimetrically using a modification of the Elson-Morgan reaction. The standard reaction mixture for determination of aminating (GlcN6P synthesizing) activity contained the following: 40 μl of 75 mM Fru-6-P and 40 μl of 1 M ammonium chloride. The main component of a standard mixture for determination of deaminating activity was 40 μl of 10-mM GlcN6P solution. Compositions of the mixtures were supplemented by appropriate amounts of the enzyme preparation (depending on the concentration and activity of the protein preparation) and buffer A (25 mM KH_2_PO_4_/K_2_HPO_4_, pH 7.0, 0.5 mM PMSF, 1 mM dithiothreitol (DTT), 1 mM EDTA) in a final volume of 400 μl. The positive control contained 40 μl of 100 mM GlcN6P and 360 μl of buffer A, and the negative control contained 400 μl of buffer A. The mixture was incubated at 37 °C for 30 min. The reaction was stopped by 1-min incubation at 100 °C, and then, the mixture was cooled on ice. To acetylate GlcN6P present in the mixture, 200 μl of saturated NaHCO_3_ solution and 100 μl of 10 % acetic anhydride in acetone prepared freshly before use were added; the mixtures were incubated at room temperature for 3 min, then for another 3 min at 100 °C and finally cooled on ice. Subsequently, 200-μl aliquots of 0.8 M K_2_B_4_O_7_ (pH 9.2–9.5) solution were added; the mixtures were boiled for 3 min and finally cooled on ice. Aliquots of the freshly prepared Elson-Morgan reagent (5 ml of solution consisted of 1 g of 4-dimethylaminobenzaldehyde, 100 ml of glacial acetic acid, and 1.5 ml of concentrated HCl) were added, and the mixtures were incubated for 30 min at 37 °C. Finally, absorbance of the samples at 585 nm was measured. One unit of specific aminating activity was defined as an amount of the enzyme that catalyzed the formation of 1 μmol of GlcN6P/min/mg protein. One unit of specific deaminating activity was defined as an amount of the enzyme that catalyzed the breakdown of 1 μmol of GlcN6P/min/mg protein.

For determination of kinetic constants, enzyme activity was determined at variable (0–10 mM) initial concentrations of appropriate substrates.

### Determination of native protein molecular mass

Oligomeric structure of recombinant GlcN6P deaminase versions was studied, and molecular masses were determined by size exclusion chromatography (SEC) on Superdex 200HR 10/300 GL column. Protein was applied in the volume of 0.5 ml and then eluted at 0.5 ml/min by 50-mM potassium phosphate buffer (pH 7.0) containing 0.15 M NaCl (buffer G). Protein elution was followed at 280 nm. The molecular mass standards were the following: dextran blue (2,000 kDa), thyroglobulin (669 kDa), β-amylase (200 kDa), alcohol dehydrogenase (150 kDa), BSA (66 kDa), and carbonic anhydrase (29 kDa). The molecular masses of the proteins studied were calculated by interpolation of a plot of log molecular mass versus *K*_av_.

The oligomeric structure was also analyzed by native PAGE using a NativPage™System with Novex® 4–16 % Bis-Tris gels kit (Invitrogen). The experiments were run according to the manufacturer’s protocol.

### Homology modeling of *Glm*NagB and its oligoHis-tagged versions

Since the real structure of the *Glm*NagB is not known so far, its hypothetical structure and those of the two oligoHis-tagged muteins were built by homology modeling. The modeling was based on the two templates: structure of the GlcN6P deaminase from *Homo sapiens* (pdbid: 1ne7) and structure of the enzyme from *B. subtilis* (pdbid: 2bkx). The amino acid sequences of these two enzymes are, respectively, 37 and 40 % identical with the sequence of *Glm*NagB. The MODELLER9v2 software (Sali and Blundell 1993) was used for comparative modeling and VMD (Humphrey et al. 1996) for visualization.

### Other methods

Protein concentration was determined by the Bradford method (1976). Discontinuous sodium dodecyl sulfate-polyacrylamide gel electrophoresis (SDS-PAGE) was performed by the method of Laemmli (1970), using a 5 % stacking gel and a 15 % separating gel.

## Results and discussion

### Cloning, expression, and purification of the recombinant proteins

Two overexpression plasmids containing DNA sequences encoding His_6_-tagged *Glm*NagB were constructed. One of them encoding fusion of *Glm*NagB with N-terminus bound His_6_-tag was obtained by cloning the *Glm*NagB gene into the pET15 vector containing the His_6_-tag encoding sequence. Another plasmid containing fusion of *Glm*NagB with C-terminal His_6_-tag addition was obtained by PCR amplification, with one of the primers introducing the oligoHis-tag encoding sequence and subsequent introduction into the Champion pET Directional TOPO vector. Plasmid containing the gene encoding the wild-type GlcN-6-P deaminase of *G. lamblia* was constructed using the Champion pET Directional TOPO vector. The sequences of plasmids were verified by sequencing.

Conditions for overexpression were optimized in terms of temperature, inductor concentration, and time profile. The overproduction results were confirmed by SDS-PAGE of cell lysates. The best overexpression was obtained in *E. coli* BL21(DE3) pLysS cells, when expression was induced by addition of the IPTG (1 mM) to the cultures grown to OD_600_≈0.5 and incubation was continued for another 5 h. Densitometric analysis revealed that in each case, the recombinant protein constituted 8–24 % of the total cytoplasmic protein pool (about 20 mg/l of the culture).

**Fig. 1:**
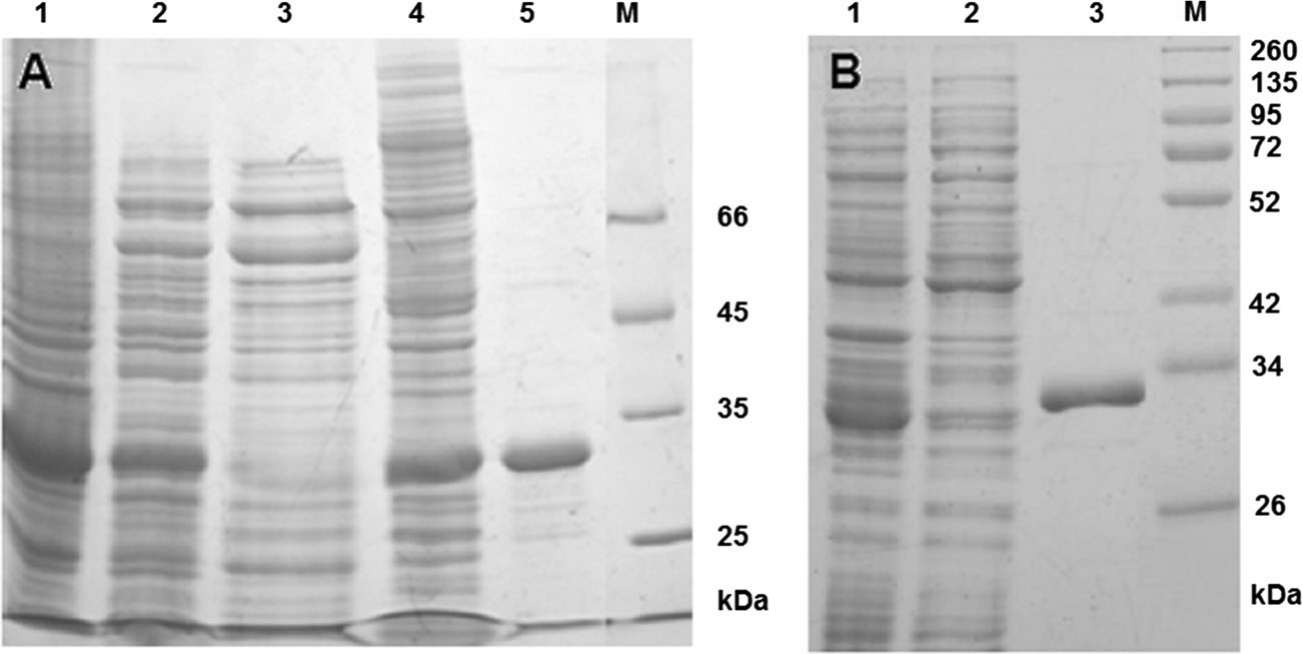
SDS-PAGE analysis of purification of recombinant *G. lamblia* GlcN6P deaminase overexpressed in *E. coli* BL21(DE3) pLysS cells. **a** Wild-type *Glm*NagB; *lane 1*—total lysate; *lane 2*—streptomycin sulfate precipitation; *lane 3*—ammonium sulfate precipitation; *lane 4*—PEG precipitation (dissolved sediment); *lane 5*—ion exchange chromatography on Resource Q; *lane M*—molecular mass markers. **b** *Glm*NagB-HisC; *lane 1*—total lysate; *lane 2*—wash of the Ni^2+^-IDA column with 0–50 mM imidazole; *lane 3*—200 mM imidazole eluate; *lane M*—molecular mass markers

The purification of the wild-type *Glm*NagB from *E. coli* BL21(DE3) pLysS cells was a multistage process. Optimization of the purification process allowed obtaining a protein preparation with a purity of 98 % with cumulative yield of 10.4 % (Fig. 1a, Table 1). Although the yield was not high, it was much better than in the procedure described by Steimle et al., where it was lower than 1 % (1997).

**Table 1:** Summary of the purification of *Glm*NagB overexpressed in *Escherichia coli* BL21(DE3) pLysS

Step	Volume [ml]	Total protein [mg]	Specific activity [U/mg]	Total activity [U]	Purification factor	Yield [%]
Crude extract	20	360	8.6	3,096	1	100
Streptomycin sulfate	22	210	13.5	2,835	1.6	91.6
Ammonium sulfate	11	65	24	1,555	2.8	50.2
PEG	9	29.2	32	936	3.7	30.2
Resource Q	6	5.2	62	322	7.2	10.4

The anabolic activity (synthesis of GlcN6P) was determined

*Glm*NagB-HisC and *Glm*NagB-HisN were purified in a single chromatographic step using the Ni^2+^-IDA agarose resin. Unbound and weakly bound proteins were washed with 0–50 mM imidazole, and the recombinant protein was eluted with 200 mM imidazole. The recombinant proteins were purified to near homogeneity (∼99 %, as revealed by a densitometric analysis) without considerable loss in protein yield. Effectiveness of purification was controlled by SDS-PAGE, and purity of the final preparations was estimated by densitometry of the CBB-stained gels. The results of the electrophoretic analysis of *Glm*NagB-HisC purification are shown in Fig. 1b. The SDS-PAGE profile for *Glm*NagB-HisN purification was apparently identical to that for *Glm*NagB-HisC (not shown). Summary of purification of the wild-type *Glm*NagB and its His_6_-tagged muteins are shown in Table 2.

**Table 2:** The final purification results of wild-type *Glm*NagB and its muteins with His_6_-tagged at C- or N-terminal

Protein	Total protein [mg/l]	Specific activity [U/mg]
*Glm*NagB	5.2	62.0
*Glm*NagB-HisC	3.2	0.5
*Glm*NagB-HisN	14.5	73.0

Anabolic activity (synthesis of GlcN6P) was determined

### Characterization of catalytic properties

The purified wild-type *Glm*NagB and its His_6_-tagged versions were characterized in terms of enzymatic activity and molecular mass. The data are summarized in Tables 2 and 3. The specific activity and kinetic parameters of the wild-type enzyme were similar to that reported in literature (Steimle et al. 1997). Data obtained for the oligo-His tagged versions indicate that introduction of the tag at either N- or C-terminus affects enzyme activity, and in the case of *Glm*NagB-HisC, it resulted in almost complete loss of catalytic properties. The aminating (GlcN6P synthesizing) activity of *Glm*NagB-HisN was almost 1.5 times higher than that of the wild-type protein, while that of *Glm*NagB-HisC was two orders of magnitude lower. Substantial changes were noted for catalytic constants. The N-terminus modified enzyme exhibited about 30 % higher *V*_max_ for the synthetic reaction than the wild-type *Glm*NagB, while the respective constant for the catabolic reaction was unchanged. A substantial decrease of *Glm*NagB-HisC affinity to GlcN6P was found, reflected by its much higher *K*_M_ for this compound as a substrate in the catabolic reaction. A slightly higher *K*_M_ for Fru6P in the anabolic reaction was also noted for this mutein. Very low values of *V*_max_ of *Glm*NagB-HisC in both reactions are the obvious consequence of the reduced activity. One may therefore conclude that catalytic properties of *Glm*NagB-HisN are similar to those of the wild-type *Glm*NagB, except for the slightly enhanced GlcN6P synthesizing activity. It is worth noting therefore that the introduced oligoHis-containing oligopeptide was quite long, as it contained 21 amino acid residues. On the other hand, introduction of a relatively small hexaHis fragment at the C-terminus resulted in a very substantial reduction of catalytic activity.

**Table 3:** Summary of catalytic properties of the wild-type *Glm*NagB and its oligoHis-tagged versions

Enzyme version	Kinetic parameters				
	Aminating direction			Deaminating direction	
	*K*_M Fru6P_ [mM]	K_M NH4Cl_ [mM]	*V*_max_ [μM/min/mg]	*K*_M GlcN6P_ [mM]	*V*_max_ [μM/min/mg]
*Glm*NagB^a^	2.5 ± 0.24	19.0 ± 1.90	86.3 ± 3.20	0.38 ± 0.16	32.8 ± 5.30
*Glm*NagB	2.8 ± 0.14	23 ± 1.20	100 ± 5.20	0.55 ± 0.05	38.1 ± 1.70
*Glm*NagB-HisN	1.71 ± 0.11	14.0 ± 0.80	128.0 ± 11.00	0.95 ± 0.15	45.2 ± 7.70
*Glm*NagB-HisC	3.52 ± 0.15	41.6 ± 3.30	2.0 ± 0.16	5.21 ± 0.40	1.21 ± 0.11

^a^Reference data (Steimle et al. 1997)

All recombinant versions of *Glm*NagB were also tested for a possible effect of GlcNAc6P, uridine 5′-diphospho-*N*-acetyl-d-glucosamine (UDP-GlcNAc), and uridine 5′-diphospho-*N*-acetyl-d-galactosamine (UDP-GalNAc) on activity. The first compound is known as an allosteric activator of *Eco*NagB (Calcagno et al. 1984) while the other two could be candidates for feedback inhibitors of the enzyme catalyzing the first committed step in the pathway of poly-GalNAc biosynthesis, by analogy to the similar role of UDP-GlcNAc, acting as a feedback inhibitor of eukaryotic GlcN6P synthase (Milewski 2002). However, in our hands, no changes in activity of *Glm*NagB were noted in the presence of any of the putative ligands at concentration ≤5 mM.

### Molecular modeling of the enzyme

**Fig. 2:**
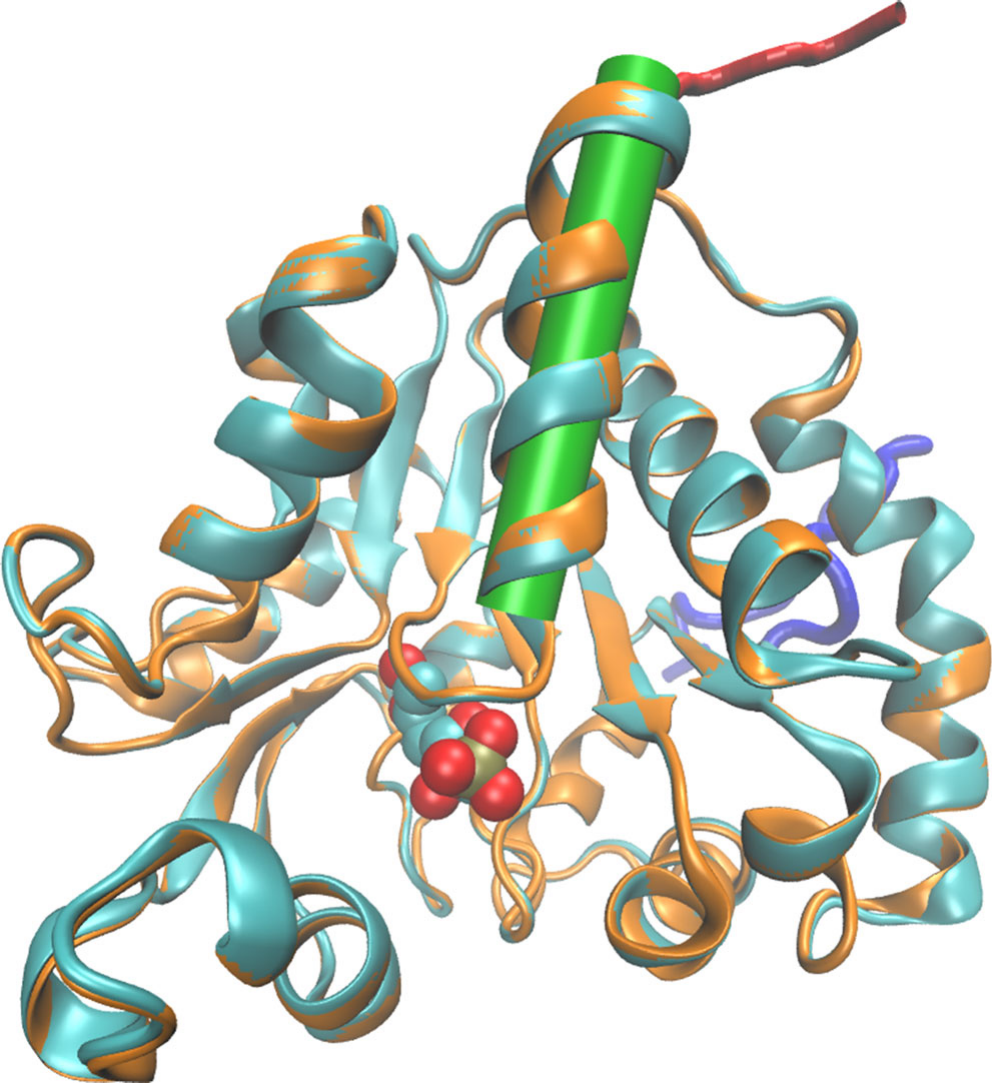
Superimposed model structures of *Glm*NagB-HisC (*orange*) and *Glm*NagB-HisN (*cyan*) with their additional oligo-His fragments, colored *red* and *blue*, respectively. The substrate F6P (transferred from the template structure 2bkx) is shown as a spacefill model bound in the active site. Location of the α1 helix is indicated by a *green* cylinder

Since the 3D structure of *Glm*NagB and its oligoHis-tagged muteins is not known, a structural basis for the observed loss of catalytic activity upon introduction of the oligoHis tag at the *Glm*NagB C-terminus is not obvious. In an attempt to clear it up, the homology models of the enzyme and two muteins were built. Superimposed images of these models are shown in Fig. 2. Its is clear that both oligoHis tags in *Glm*NagB-HisC as well as in *Glm*NagB-HisN are located far from the active site; thus, they are not able to interfere with it directly. However, whereas the longer N-terminal fragment present in *Glm*NagB-HisN forms irregular loop at the site opposite to the active center, the shorter C-terminal oligo-His fragment of *Glm*NagB-HisC is located close to the C-terminal part of the α1 helix. This helix, particularly its N-terminal part, reach in Ser and Thr residues and containing conserved Gly36, corresponds to the analogous structure existing in all known structures of GlcN6P deaminases, which is directly involved in binding of the Fru6P or GlcN6P phosphate group and comprises common structural motif existing in various enzymes containing phosphate binding sites (Copley and Barton 1994). Preliminary molecular dynamics calculations (details not shown) revealed that the introduced C-terminal oligo-His fragment is possibly capping the C-terminal part of the α1 helix, thus interacting with it directly and affecting its dipole moment what in consequence may influence the substrate binding.

### Determination of quaternary structure

**Fig. 3:**
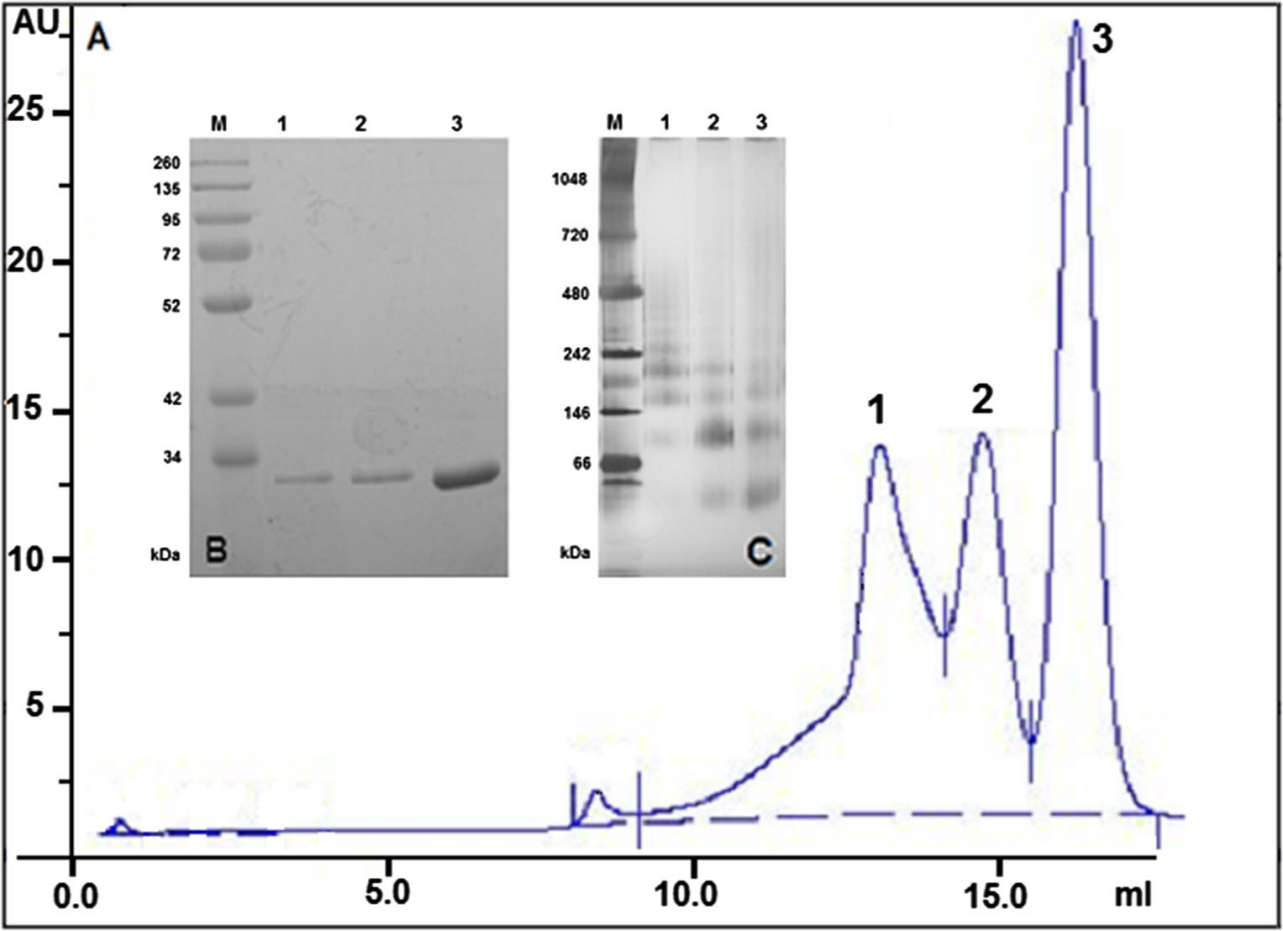
**a** Results of SEC analysis of native *Glm*NagB on Superdex 200HR 10/300 GL column. *AU* arbitrary units; Inside, **b** SDS-PAGE analysis of fractions collected from SEC; **c** Native-PAGE analysis of fractions collected from SEC. *Lane M*—molecular mass markers; *lanes 1, 2 and 3*—fractions indicated in **a**

SDS-PAGE analysis of *Glm*NagB, *Glm*NagB-HisN, and *Glm*NagB-HisC revealed presence of single bands migrating at 30.5 ± 1.0, 32.5 ± 1.0, and 32.0 ± 1.0 kDa, respectively. These values are in a good agreement with theoretical molecular masses calculated for the appropriate amino acid sequences: 29.4 kDa for *Glm*NagB, 31.7 kDa for *Glm*NagB-HisN, and 30.2 kDa for *Glm*NagB-HisC (calculated by Compute pI/MW). In contrast, multiple forms of *Glm*NagB and its oligoHis-tagged versions were found in SEC experiments. In all cases, three peaks corresponding to proteins of different sizes were detected (Fig. 3a, an exemplary SEC profile of the wild-type enzyme). Samples collected from respective fractions were analyzed for enzyme activity, effect of GlcNAc6P, UDP-GlcNAc, and UDP-GalNAc, by SDS-PAGE and by native PAGE. Results of these analyzes are shown in Table 4 and Fig. 3b, c. They showed that each of the enzyme versions occurred in three oligomeric forms, namely, monomeric, homodimeric, and homotetrameric. Oligomerization did not affect enzyme-specific activity, which was very similar for all the forms. None of the forms was activated/inhibited by GlcNAc6P, UDP-GlcNAc, or UDP-GalNAc. The relative content of oligomeric forms in analyzed samples was variable and depended mostly on protein concentration and storage period. The monomeric form was always dominant, but the monomer/dimer/tetramer ratios fell within the range from 72:27:1 to 39:23:38. The former ratio was characteristic for fresh protein preparations, obtained immediately after the purification process. The latter ratio was observed for preparations stored for several hours at 4 °C and for preparations with higher protein concentration. Interestingly, in samples of particular fractions from the SEC separation, containing a homogenous quaternary form of the enzyme, the other two forms were formed upon storage, with the whole system obviously going toward equilibrium. This tendency is also clear when looking at the results of native-PAGE analysis of fractions collected from SEC (Fig. 3c). Although in each of the lanes 1–3, one of the bands is predominant, the other ones are also present, and this is likely a consequence of equilibration occurring in samples collected from SEC and stored for further native-PAGE analysis. Notably, the molecular masses calculated from the locations of the bands present in Fig. 3c are higher than the values expected for the monomer, dimer, or tetramer of *Glm*NagB (48, 95, 201 kDa), but similar deviations from the expected MWs have been reported previously for results of the native-PAGE analysis of other proteins, for example, fungal homocitrate synthase (Schöbel et al. 2010).

**Table 4:** Summary of results of SEC analysis of the wild-type *Glm*NagB and its oligoHis-tagged muteins

Fraction	Native MW (SEC) [kDa]	MW of the denatured protein (from SDS-PAGE) [kDa]	Specific activity [U/mg]	Putative quaternary structure
*Glm*NagB				
1	131.4 ± 25.0	30.7 ± 2.0	48	Homotetramer
2	58.0 ± 11.0	30.7 ± 2.0	55	Homodimer
3	27.7 ± 5.0	30.7 ± 2.0	51	Monomer
*Glm*NagB-HisN				
1	121.0 ± 24.0	33.0 ± 2.0	97	Homotetramer
2	69.4 ± 14.0	32.6 ± 2.0	89	Homodimer
3	31.1 ± 6.0	32.6 ± 2.0	80	Monomer
*Glm*NagB-HisC				
1	138.7 ± 27.0	32.0 ± 2.0	0.48	Homotetramer
2	59.4 ± 11.0	32.0 ± 2.0	0.50	Homodimer
3	28.4 ± 5.0	32.0 ± 2.0	0.52	Monomer

**Fig. 4:**
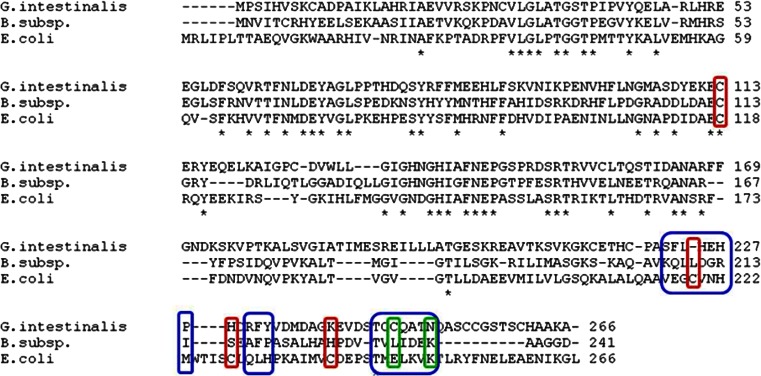
Multiple alignment of amino acid sequences of glucosamine-6-phosphate deaminase from *Giardia intestinalis*, *Bacillus* subsp., and *Escherichia coli*. Residues responsible for the formation of the hexameric structure of *E*coNagB and their counterparts in *Giardia* and *Bacillus* proteins are in the *blue frames*. Glu246 and Lys250 which form a salt bridge linking a dimer of trimers in *E*coNagB and their counterparts in *Giardia* and *Bacillus* proteins are in *green frames. In red frames*, the Cys residues that were supposed to be involved in disulfide bridges in *E*coNagB and their counterparts in other enzyme versions

Results of the multiple structural alignment of *Giardia*, *Bacillus*, and *E. coli* GlcN6P deaminase shown in Fig. 4 clearly indicate that the residues 216–223, 230–232, and 244–250, identified as those responsible for the formation of the hexameric structure of *Eco*NagB (Oliva et al. 1995), have no counterparts of similar character in two other enzyme versions. Moreover, out of the four cysteinyl residues suspected to be involved in formation of intersubunit disulfide bonds in *Eco*NagB, namely, Cys118, Cys219, Cys228, and Cys239 (Altamirano et al. 1992, 1993; Horjales et al. 1992), only the first one is conserved in *Glm*NagB (Fig. 4). It is well understandable therefore why *Glm*NagB is not hexameric, despite the relatively high homology of *Glm*NagB and *Eco*NagB sequences (41 % identity). On the other hand, an amino acid sequence of *Glm*NagB exhibits similar homology to those of the monomeric GlcN6P deaminase from *B. subtilis* (40 %) but lower to the dimeric NagB from *Thermococcus kodakaraensis* (25,1 %). Since NagB from archeons is homologous rather to the isomerase domain of GlcN6P synthase (ISOM) than to other NagBs from mesophilic organisms (Kim et al. 2007; Tanaka et al. 2005), it is obvious that *Glm*NagB is not similar to ISOM. The amino acid sequence of *Glm*NagB is homologous to that of GlcN6P deaminase of human pathogenic yeast *Candida albicans* encoded by the *NAG1* gene (41 % identity), which is not activated by GlcNAc6P and supposed to be dimeric in the native form (Natarajan and Datta 1993), but this supposition has not been unequivocally confirmed yet. However, the GlcN6P deaminase of *C. albicans* is a catabolic enzyme, similarly as most of the representatives of the NagB subfamily, so that one cannot expect any close structural and functional analogy between these two enzyme versions.

The structural alignment reveals also a possible reason for the lack of an activating effect of GlcNAc6P on *Glm*NagB. In *Eco*NagB, seven amino acid residues were identified as those directly involved in binding of the allosteric activator, namely, Tyr121, Asp141, Glu148, Ser151, Arg158, Lys160, and Tyr254 (Oliva et al. 1995; Vincent et al. 2005). Only four of them are conserved in *Glm*NagB, as Tyr116, Asp143, Ser146, and Arg153, respectively.

Due to the heterogeneity of its quaternary structure, *Glm*NagB may belong to the class of proteins known as morpheeins. The known examples include *Pseudomonas aeruginosa* GDP-mannose dehydrogenase (EC 1.1.1.132), mammalian CoA transferase (EC 2.8.3.5), and plant porphobilinogen synthase (EC 4.2.1.24) (Jaffe 2005). However, in the case of such protein, their oligomerization is one of the ways of activity regulation, and their oligomeric forms differ in activity and/or sensitivity to allosteric regulators. This seems not be the case for *Glm*NagB, so that any functional reason for the observed heterogeneity of its quaternary structure is not known.

## Conclusions

The oligoHis-tagged versions of *Glm*NagB constructed and studied in this work demonstrate the same type of heterogeneity of quaternary structure as the wild-type protein, but the catalytic properties of *Glm*NagB-HisC are substantially different from those of *Glm*NagB-HisN and *Glm*NagB. Introduction of the hexaHis tag at the C-terminal of *Glm*NagB strongly reduces GlcN6P synthetic activity of the enzyme, while presence of the tag at N-terminus has little if any effect. Apparent identity of kinetic parameters, insensitivity to the action of possible allosteric effectors (GlcNAc6P, UDP-GlcNAc, and UDP-GalNAc) and existence in solution in three interconvertible quaternary forms (monomer, dimer, and tetramer) indicate that the easily isolable *Glm*NagB-HisN may serve as an adequate model of the wild-type enzyme in all structural studies.

*Glm*NagB is obviously different from other GlcN6P deaminases known so far, due to its dominant anabolic, not catabolic function, and the unique quaternary structure heterogeneity. There is little doubt therefore that *Glm*NagB enzyme represents the fourth molecular version of enzymes of the NagB subfamily. Further, more detailed structural and mechanistic studies on this enzyme are highly desirable due to its potential as a putative target for antiprotozoal chemotherapy.
